# MicroRNA155 in non-small cell lung cancer: a potential therapeutic target

**DOI:** 10.3389/fonc.2025.1517995

**Published:** 2025-02-03

**Authors:** Xiangju Wei, Xianmin Xiong, Ze Chen, Bi Chen, Cantang Zhang, Wenhui Zhang

**Affiliations:** ^1^ The First Clinical College, Xuzhou Medical University, Xuzhou, China; ^2^ Department of Respiratory and Critical Care Medicine, The Affiliated Hospital of Xuzhou Medical University, Xuzhou, China

**Keywords:** microRNA, microRNA155, non-small cell lung cancer, immune response, oncogenic

## Abstract

Lung cancer (LC) is the second most commonly diagnosed cancer among both men and women, and it stands as the leading cause of cancer-related mortality, characterized by high rates of morbidity and mortality. Among its subtypes, non-small cell lung cancer (NSCLC) is the most prevalent and one of the most challenging malignant tumors to treat. To date, various therapeutic approaches, including surgery, radiotherapy, and chemotherapy, have been employed in the management of lung cancer; however, due to its aggressive nature, the survival rates remain low. Consequently, exploring novel treatment strategies is of paramount importance. MicroRNAs (miRNAs), a large family of non-coding RNAs, play crucial roles in regulating several key biological processes, including cell proliferation, differentiation, inflammation, and apoptosis. Among these, microRNA155(miR-155) is one of the most conserved and versatile miRNAs, predominantly overexpressed in various diseases, including malignant tumors. This review elucidates the biological functions and roles of miR-155 in NSCLC and discusses its potential significance as a therapeutic target for future research directions and clinical applications.

## 1 Introduction

**Figure 1:**
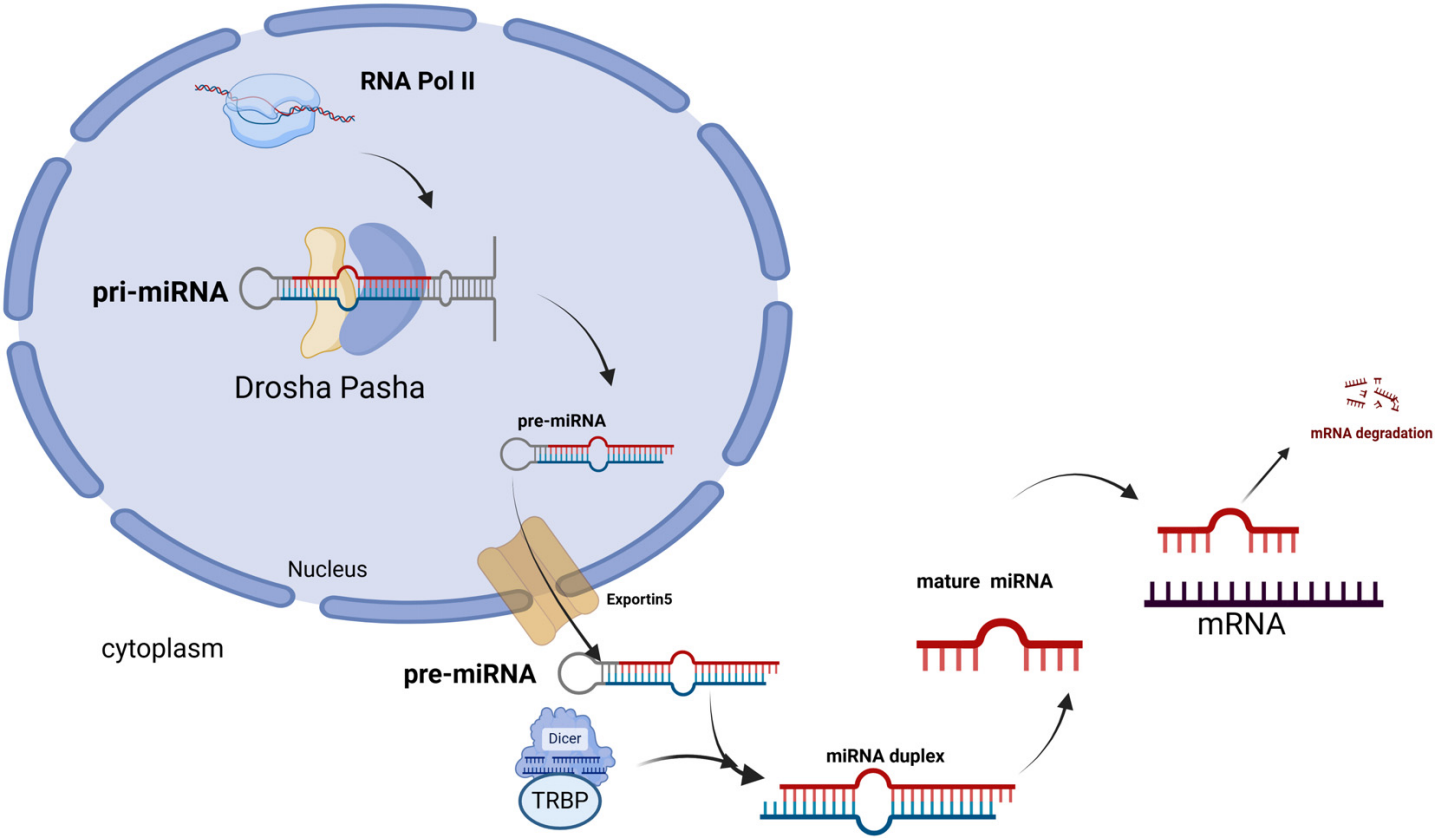
In the nucleus, primary miRNA is synthesized by RNA polymerase II, and these molecules are subsequently cleaved by the RNase III enzyme Drosha, along with its cofactor DiGeorge syndrome critical region 8 (DGCR8, also known as Pasha), to generate single-stranded RNA precursors. These precursors are then transported into the cytoplasm by the nuclear export protein Exportin 5. In the cytoplasm, the loop region of pre-miRNA is cleaved by the RNase III enzyme Dicer and the transactivation response element RNA-binding protein (TRBP), resulting in the formation of a miRNA duplex. Following the discarding of one strand, mature miRNA is produced. Finally, mature miRNA binds to the 3’ untranslated region (3’ UTR) of the target mRNA, which leads to mRNA degradation.

Lung cancer is the leading cause of cancer-related deaths worldwide, characterized by high morbidity and mortality rates. In 2021, there were an estimated 2.3 million confirmed cases and 1.8 million deaths. Based on comprehensive staging, the current five-year relative survival rate is approximately 22%. Non-small cell lung cancer (NSCLC) is the most prevalent histological type, and most cases are diagnosed at an advanced stage. Comprehensive treatment options, including surgery, chemotherapy, and radiotherapy, have been employed for lung cancer management; however, the therapeutic outcomes remain unsatisfactory. The emergence of drug resistance in current lung cancer treatments underscores the urgent need to explore new therapeutic avenues (1–5). Recent epigenetic advancements have opened new pathways for understanding the mechanisms and potential therapeutic targets associated with NSCLC progression and metastasis (6, 7). MiRNAs are a class of endogenous non-coding small RNAs, ranging from 18 to 24 nucleotides in length. Within the nucleus, Drosha, in conjunction with DGCR8 (also known as Pasha), first cleaves the primary miRNA (pri-miRNA) into a miRNA precursor (pre-miRNA) of approximately 70 nucleotides. This precursor is then transported to the cytoplasm via the nuclear export protein Exportin 5(Exportin 5). In the cytoplasm, the loop region of the miRNA precursor is removed by Dicer RNase III and the transactivation response element RNA-binding protein (TRBP), resulting in the formation of a miRNA duplex, from which one strand is discarded to produce a mature miRNA (8–10). Ultimately, by binding to the 3’ untranslated region (3’ UTR) of the target mRNA, miRNAs reduce the levels of proteins encoded by the target mRNA through mechanisms such as mRNA degradation and inhibition of mRNA translation. Consequently, miRNAs regulate gene expression via translation inhibition, mRNA cleavage, and rapid deadenylation of mRNA (11) (See **Figure 1**).

As a member of the microRNA family, miR-155 is processed from the exons of a non-coding RNA known as the B-cell Integration Cluster (BIC) and exhibits strong sequence homology across mammals, indicating that its function is evolutionarily conserved (12). Increasing evidence suggests that miR-155 possesses a distinct expression profile and is involved in various physiological and pathological processes, including cancer, inflammation, hematopoiesis, and immunity (13).

Several studies have indicated that, in addition to its abnormal expression in NSCLC, miR-155 is also implicated in the differential expression observed in various other cancers. This characteristic allows miR-155 to effectively differentiate tumor tissues from normal tissues based on expression levels. For instance, its expression is notably upregulated in a range of human malignancies, including breast cancer, pancreatic cancer, and colon cancer, as well as in B-cell lymphoma and chronic lymphocytic leukemia (14, 15). Conversely, its expression is downregulated in esophageal cancer (which is associated with a reduced immune response), gastric cancer (16) (where it inhibits gastric cancer cell proliferation and promotes cell apoptosis), and ovarian cancer (where it down-regulates Claudin-1), among others.

In summary, the dysregulation of miR-155 holds significant implications for cancer. Regulating miR-155 expression has emerged as a promising approach for cancer treatment, and investigating the role of miR-155 in the pathogenesis of NSCLC will enhance our understanding. Identifying and targeting key genes involved in NSCLC metastasis and treatment will be crucial for future therapeutic strategies. This study discusses the potential impact of these findings on the clinical management of NSCLC and their relevance to clinical treatment (17–19).

## 2 Subtypes of lung cancer

**Figure 2:**
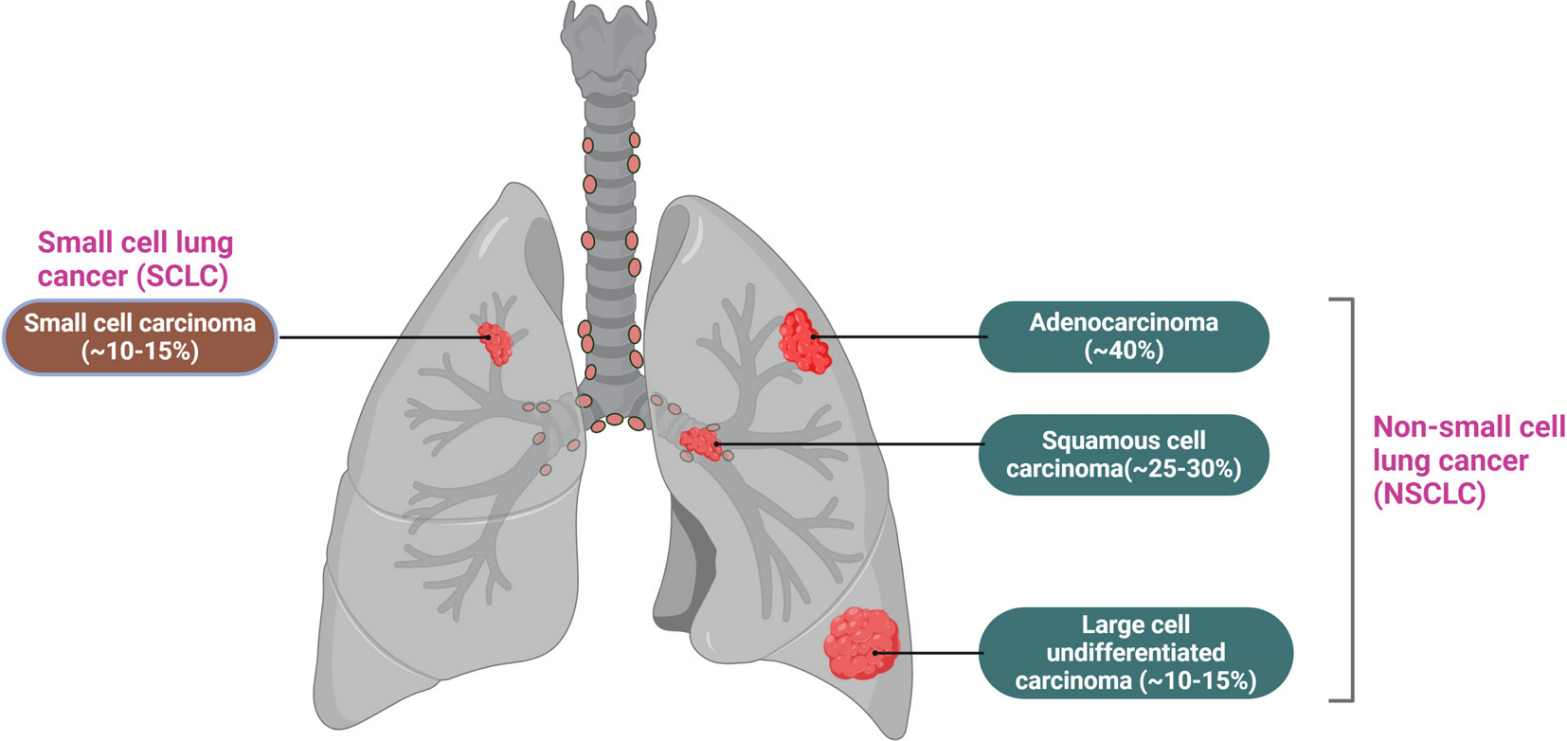
Lung cancer is mainly divided into two types: small cell lung cancer, which accounts for 15%, and non-small cell lung cancer, which accounts for 85%. Among non-small cell lung cancer, it is further classified into adenocarcinoma, squamous cell carcinoma, and large cell undifferentiated carcinoma, accounting for 40%, 25-30%, and 10-15%, respectively.

Lung cancer is primarily categorized into two types (See **Figure 2**): non-small cell lung cancer (NSCLC), which accounts for approximately 85% of all lung cancer cases, and small cell lung cancer (SCLC), comprising the remaining 15% (20). NSCLC is the most prevalent subtype of lung cancer and is recognized as one of the most challenging malignancies to treat. This subtype is further classified into three histological categories: adenocarcinoma (AC, LUAD), which represents around 40%; squamous cell carcinoma (SCC, LUSC), accounting for 30%; and large cell carcinoma (LCC), which constitutes about 10% (21). The pathogenesis of NSCLC is highly complex and involves the impaired activation of multiple intracellular signaling pathways, including the phosphatidylinositol 3-kinase (PI3K),mitogen-activated protein kinase (MAPK), and epidermal growth factor receptor (EGFR) pathways. Additionally, mutations in several genes, such as phosphatidylinositol 3-kinase catalytic alpha (PIK3CA), phosphatase and tensin homolog (PTEN), and Kirsten rat sarcoma viral oncogene homolog (KRAS), play a significant role in NSCLC development (22). Furthermore, research indicates that the progression of NSCLC is influenced by the tumor microenvironment (TME) (23), which encompasses the tumor itself, adjacent immune cells, fibroblasts, vascular endothelial cells, extracellular matrix components, and various cytokines. The TME significantly promotes tumor growth, suppresses immune responses, and facilitates tumor invasion and metastasis (24, 25). Despite ongoing advancements in lung cancer treatments and a decline in incidence rates, lung tumors remain a leading cause of death and morbidity worldwide due to late diagnosis and poor prognostic outcomes.

## 3 Role of microRNAs in cancer

MicroRNAs (miRNAs) can act as either oncogenes or tumor suppressor genes, thus playing a crucial role in regulating tumor progression. A growing body of evidence indicates that miRNAs are significantly involved in various aspects of tumor biology, including proliferation, inhibition, diagnosis, prognosis, and metastasis (26, 27).

**Figure 3:**
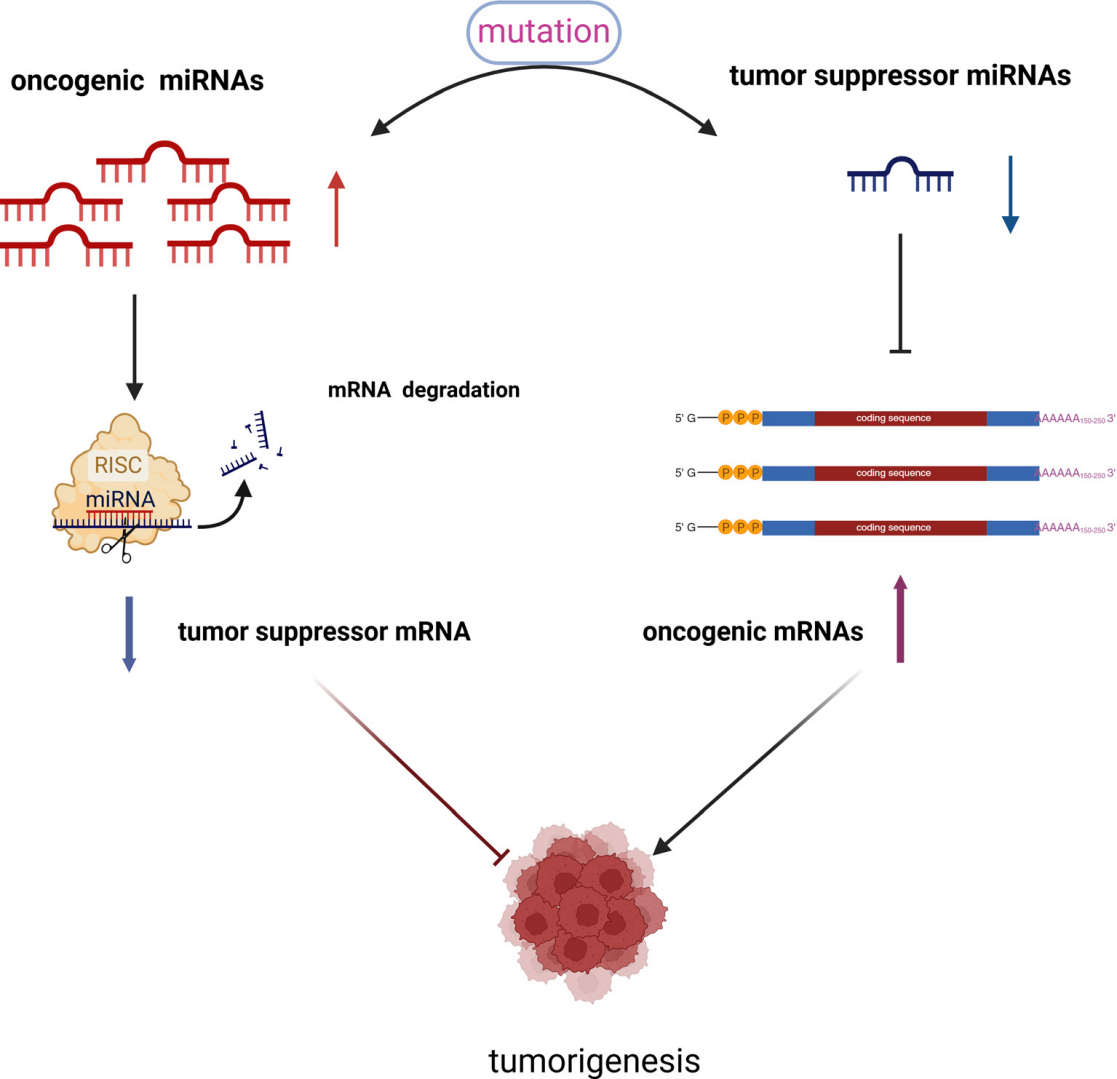
There exists a competitive relationship between oncogenic miRNAs and tumor suppressor miRNAs. When the expression levels of oncogenic miRNAs increase in cells, the expression of certain tumor suppressor genes is inhibited, which occurs through mechanisms such as mRNA degradation or hypermethylation. Consequently, the expression of tumor suppressor miRNAs decreases. Conversely, when the expression of tumor suppressor miRNAs is reduced, the expression of oncogenic miRNAs increases. It is through this interplay that oncogenic miRNAs and tumor suppressor miRNAs collaboratively contribute to the occurrence and progression of tumors.

Research by Lu et al. (28) demonstrated that the expression patterns of a specific group of cellular miRNAs are altered in cancerous tissues compared to normal tissues. Furthermore, the expression profiles of these miRNAs are associated with both the tumor’s origin and, notably, its differentiation stage. Dysregulation of miRNA expression in cancer can occur through four primary mechanisms: chromosomal abnormalities, genomic mutations and polymorphisms, epigenetic alterations, and disruptions in miRNA biosynthesis (29). Based on their biological roles in cancer, miRNAs can be broadly classified into two categories: tumor suppressor miRNAs and oncogenic miRNAs (See **Figure 3**). Tumor suppressor miRNAs typically target cellular oncogenes and are often down-regulated in cancer. An example is the let-7 family in lung cancer (30), which inhibits various oncogenes such as Rat Sarcoma (RAS), Myelocytomatosis Viral Oncogene Homolog (MYC), and High Mobility Group AT-hook 2 (HMGA2), thereby reducing the expression of cell cycle proteins (31–35). Another tumor suppressor miRNA, miR-34a, targets oncogenes including MYC and Bcl-2-Associated X Protein(Bax) (36).In contrast, oncogenic miRNAs, such as miR-21, target tumor suppressor genes like Phosphatase and Tensin Homolog (PTEN) and Programmed Cell Death 4 (PDCD4). Following this classification, therapeutic strategies based on miRNAs can be divided into two major subgroups: replacement of the loss of specific miRNAs using miRNA restoration agents such as miRNA mimics, and blocking aberrantly overexpressed miRNAs using inhibiting agents, such as antagomiRs (anti-miRs), miRNA sponges and target protectors (37, 38). In summary, miRNAs play a crucial role in regulating various cellular processes involved in cancer progression. Given their ability to target multiple oncogenic or tumor suppressor signaling pathways, miRNAs hold significant potential as therapeutic agents (39–42).

### 3.1 Role of microRNAs in NSCLC pathogenesis

In recent decades, numerous studies have established that miRNAs function as either tumor suppressors or oncogenic factors, influencing processes from the initial uncontrolled growth of tumor cells to metastasis and angiogenesis. This regulatory role primarily occurs through the modulation of tumor-related signaling pathways (23). Dysregulation of miRNAs can lead to the development of various diseases, including cancer, neurological disorders, cardiovascular diseases, single-gene disorders, and autoimmune diseases, all of which have been linked to the disruption of miRNA function (34). MiRNA plays a significant role in the pathogenesis of NSCLC. These small non-coding RNA molecules are involved in the regulation of gene expression at the post-transcriptional level by binding to the 3’ untranslated region (3’ UTR) of target mRNA, which results in mRNA degradation or translational inhibition. In NSCLC, the dysregulation of miRNAs contributes to various stages of cancer development, including initiation, progression, metastasis, and drug resistance. Key miRNAs associated with NSCLC include miR-21 (43, 44), miR-126 (45, 46), miR-155 (47), miR-146a (20), miR-210 and miR-486 (48, 49), miR-182,miR-183 and miR-210 (50), miR-10b and miR-145 (51), miR-340 (52), miR-133b (53), miR-132 (24), miR-106a (25), miR-152 (26), miR- 195 (28), miR- 378 (29), miR- 6713p and miR- 646 (30, 31), miR-223 (54–57), miR-489-3p (58), miR-6884-5p (59), miR-522-3p (60)(See **Table 1**).

**Table 1:** Details of various miRNAs reported to be associated with NSCLC.

miRNAs	Role inNSCLC	Experimental Methods/Objectives	Important Findings/Results	Ref.
miR-21	Diagnosis OncomiR	Developed a molecular beacon (MB)-based miRNA detection strategy for NSCLC. The effect of miR-21 on PTEN expression was assessed in NSCLC cell lines with miR-21 inhibitor to decrease miR-21 expression.	MiR-21 in NSCLC PBMCs as a possible biomarker. miR-21 post-transcriptionally down-regulates the expression of tumor suppressor PTEN and stimulates growth and invasion in NSCLC.	(43, 44)
miR-126	Diagnosis Prognosis	To estimate the diagnostic and prognostic value of miR-126 in lung cancer.	Shown that miR-126 can regulate the induction and function of CD4+Foxp3+ regulatory T cells through the PI3K/AKT pathway.	(45, 46, 61)
miR-155	OncomiR	To investigate the therapeutic effects of nanoparticle-delivered anti-miR-155 in NSCLC, alone or in combination with standard-of-care drugs.	It bridges the gap between preclinical development and clinical translation of anti-miR-155 and unravels the potential of anti-miR-155 combination therapies in NSCLC.	(47)
miR-146a	Diagnosis Tumor suppressor	To investigate the expression of miR-146a and miR-155 linked to blood immune cell phenotypes and serum cytokines in NSCLC patients.	The miR-146a expression in PBMC and serum TGF-b and IL-1b levels may act as blood markers in NSCLC patients. miR-146a has a major effect on programmed cell death, and its overexpression suppresses cell migration and proliferation in NSCLC cell lines	(20)
miR-210 miR-486	Diagnosis	To evaluate the potential values of miRNAs as plasma biomarkers for early diagnosis in NSCLC by comparing them with other typical plasma biomarkers.	MiR-486 and miR-210 could be potential blood-based biomarkers for early diagnosis of NSCLC.	(48, 49)
miR-182 miR-183 miR-210	Diagnosis	To investigate the serum levels of four different miRNAs in patients with NSCLC and assess their diagnostic value for NSCLC.	MiR-182, miR-183, and miR-210 alone were useful as tumor biomarkers for the early detection of NSCLC.	(50)
miR-10b miR-145	Metastasis	Expression of both miR-145 and miR-10b was correlated with lymph node metastasis in NSCLC.	MiR-10b and miR-145 may act as an oncogene or tumor suppressor gene, respectively, in NSCLC metastasis.	(51)
miR-340	Tumor suppressor	Demonstrated that CDK4 was a direct target of miR-340 in NSCLC cells and miR-340 suppressed cell proliferation by targeting the 3’UTR of CDK4 *in vitro* and *in vivo*	Revealed that miR-340 functions as a tumor suppressor in NSCLC cells and may provide a potential target for NSCLC treatment.	(52)
miR-133b	Tumor suppressor	Determined the anticancer activity and its mechanism of miR-133b on cell proliferation of cisplatin-induced non-small cell lung cancer cells	Over-regulation of miR-133b inhibits cell proliferation of cisplatin-induced NSCLC by PI3K/Akt and JAK2/STAT3 signaling pathway by targeting EGFR.	(53)
miR-132	Tumor suppressor	To investigate the putative role of miR-132 in metastasis of NSCLC.	MiR-132 suppresses the migration and invasion of NSCLC cells by targeting zinc finger E-box binding homeobox 2(ZEB2) involving the EMT process.	(62)
miR-106a	Tumor suppressor	To investigate the expression and the biological roles of miR-106a in non-small cell lung cancer.	MiR-106a inhibited the growth and metastasis of NSCLC cells by decreasing PTEN expression.	(26)
miR-152	Tumor suppressor Prognostic	Overexpression of miR-152 suppressed cell proliferation and colony formation and also limited migration and invasion	MiR-152 suppressed the proliferation and invasion of NSCLC cells by downregulating FGF2.	(63)
miR-195	Tumor suppressor	Overexpression of HDGF dramatically abolished the tumor suppressive role of miR-195 in NSCLC cells.	A tumor suppressive role of miR-195 in NSCLC and suggested a potential therapeutic target for NSCLC.	(64)
miR-378	Tumor suppressor	To investigate whether HMOX1 can modulate miRNAs and regulate human NSCLC development.	The interplay between HMOX1 and miR-378 significantly modulates NSCLC progression and angiogenesis, suggesting miR-378 as a new therapeutic target.	(65)
miR-671-3p miR- 646	Tumor suppressor	MiR−671−3p was significantly upregulated in NSCLC tissues compared with adjacent normal tissues. Overexpression of miR-646 could suppress NSCLC cell proliferation, clonogenicity, and invasion, and inhibit epithelial-mesenchymal transition (EMT)	MiR-671-3p and miR-646 can both target cyclin D2(CCND2) mRNA, reducing CCND2 levels and thereby inhibiting the proliferation and invasion of NSCLC cells.	(66, 67)
miR-223	Diagnosis Tumor suppressor	Used ddPCR technology to validate miR-223 as a robustly measurable serum biomarker of early-stage NSCLC. To further explore the molecular mechanisms of miRNA-223 in non-small cell lung cancer (NSCLC).	Serum miR-223 was validated as an effective biomarker of stage I-II NSCLC, confirming the miR-223 diagnostic performance reported by others in Chinese cohorts. MiR-223 may induce the apoptosis of NSCLC through the PI3K/AKT pathway by EGFR.	(54–57)
miR-489-3p	Tumor suppressor	To investigate the molecular mechanism of miR-489–3p in NSCLC.	MiR-489–3p overexpression may inhibit NSCLC cell proliferation and migration by suppressing the HER2/PI3K/AKT/Snail signaling pathway	(58)
miR-6884-5p	Tumor suppressor Treatment	Used miR-6884-5p mimics and inhibitors to assess its effects in NSCLC.miR-6884-5p expression levels in NSCLC cell lines were quantified using qRT-PCR.	MiR-6884-5p effectively suppressed transforming growth factor β1-induced epithelial-mesenchymal transition, as evidenced by the restored expression of E-cadherin, N-cadherin, and Vimentin leading to the inhibition of migration and invasion in NSCLC cell lines.	(59)
miR-522-3p	brain metastasis	To investigate the role of miR-522-3p in the development of NSCLC brain metastasis.	The findings confirmed that miR-522-3p and TNS1 can affect BBB permeability by regulating ZO-1 and OCLN expression.	(60)

## 4 Role of microRNA155 in cancer

Many recent studies have demonstrated that miR-155 plays a significant role in the occurrence and progression of various solid tumors as well as hematological malignancies. For instance, In breast cancer tissues and plasma or serum, the expression of miR-155 is elevated and shows a negative correlation with the levels of estrogen receptor (ER) and progesterone receptor (PR) (11). Peng et al. suggested that miR-155 promotes bladder cancer growth by inhibiting the tumor suppressor DMTF1 (68). Additionally, Al-Haidari et al. found that miR-155-5p positively regulates CCL17-induced colon cancer by targeting RhoA, thereby enhancing cell migration (69). Fu et al. posited that miR-155-5p inhibits PTEN through the AKT signaling pathway, promoting the progression of hepatocellular carcinoma (70). Lei et al. indicated that miR-155 is involved in glioma progression by regulating the expression and function of the caudal-type homeobox 1(CDX1) (71). Furthermore, Charles H et al. observed that the expression levels of miR-155 and miR-21 in activated B cells were higher than those in biochemical center B cells (72). Li et al.indicated that miR-155-5p regulated the development of cervical cancer cells by regulating the expression of TP53INP1 (73). Several researchers have also discovered that by targeting the cytokine signaling inhibitor the suppressor of Cytokine Signaling 1(SOCS1), they regulate STAT3-related signaling pathways, ultimately leading to the occurrence and development of cancer (74–76). In 2018, Seto et al. demonstrated that miR-155 simultaneously regulates multiple parallel survival pathways associated with the pathogenesis of mycosis fungoides, including the JAK/STAT, MAPK/ERK, and PI3K/AKT pathways (77). Besides, miR-155 promotes proliferation and inhibits apoptosis of nasopharyngeal carcinoma cells through targeting the PTEN-PI3K/AKT pathway (78). In summary, miR-155 is implicated in the occurrence and progression of various cancers, which suggests its potential utility in exploring novel cancer treatments. While the abnormal expression of miR-155 can serve as a marker for cancer diagnosis and prognosis, as well as an important target for therapy, it is crucial to consider the differential mechanisms of miR-155 across different tumor types. Therefore, we propose that future studies should adopt a combined targeting strategy based on tumor-specific biomarkers, which may enhance the therapeutic effects of anti-miR-155 therapies in conjunction with immunotherapy (79).

### 4.1 MicroRNA155 regulates multiple cellular processes associated with NSCLC pathogenesis

MiR-155 is one of the most extensively studied microRNA (miRNA) molecules, with its abnormal expression implicated in a variety of pathological processes. Research indicates that its expression levels regulate pathways associated with fundamental cellular functions, including cell proliferation, invasion, migration, metastasis, drug resistance, and immune responses. Given its broad regulatory potential in key cellular mechanisms in NSCLC, miR-155 is particularly promising for both research and clinical applications (78, 80, 81).

#### 4.1.1 Role of MiR-155 in NSCLC proliferation

MiR-155 is one of the most conserved and versatile microRNAs, primarily overexpressed in various diseases, including malignant tumors. The overexpression of miR-155 may enhance cell proliferation by downregulating the cell cycle regulator WEE1 and tumor protein 53-induced nuclear protein 1 (TP53INP1). This downregulation can increase the mutation rate by targeting core components of the DNA mismatch repair mechanism, ultimately leading to the genesis of cancer. A study by Hou et al. demonstrated that the expression of miR-155 is elevated in NSCLC tissues and cell lines. The increased levels of miR-155 significantly enhanced the proliferation of A549 cells, reduced the S phase cell population (the DNA replication phase), and increased the G2/M phase cell population (mitosis) (82). Conversely, downregulation of miR-155 expression significantly inhibited cell proliferation, increased the proportion of cells in the S phase, and decreased the proportion of cells in the G2/M phase. Additionally, miR-155 promotes NSCLC cell proliferation through FOXO1 and increases the production of reactive oxygen species (ROS) (83). Coincidentally, research by Liu et al. also revealed that miR-155 inhibits proliferation and invasion in NSCLC by directly targeting PDCD4. The expression of the 64-kDa protein PDCD4 is significantly downregulated in various cancers, including colorectal, lung, gastric, and breast cancers, and is thus generally regarded as an important tumor suppressor (84). A recent study has, for the first time, demonstrated the high expression of miR-155-5p and the low expression of Fibroblast Growth Factor 9(FGF9) in lung squamous cell carcinoma tissues and cells, revealing a negative correlation between the two. Molecular mechanism experiments indicate that miR-155-5p promotes the proliferation and invasion of lung squamous cell carcinoma cells by targeting and negatively regulating FGF9 (85). Additionally, studies by Xue et al. have demonstrated that the Suppressor of Cytokine Signaling 1 (SOCS1) (86), Suppressor of Cytokine Signaling 6 (SOCS6) (87), and Phosphatase and Tensin Homolog (PTEN) (88) are implicated in various human malignant tumors, including NSCLC. Identified as tumor suppressors, miR-21 and miR-155 promote the development of NSCLC by downregulating SOCS1, SOCS6, and PTEN.

#### 4.1.2 Role of MiR-155 in NSCLC migration, invasion and metastasis

MiR-155-5p refers to the mature strand of miR-155, specifically the 5’strand that is processed following the transcription of this gene. MicroRNAs typically exist as two strands: the 5’strand and the 3’ strand. miR-155-5p, being the mature form of the 5’chain, inhibits the migration and invasion of lung adenocarcinoma A549 cells by targeting Smad2 (89).Furthermore, it is posited that the activation of Smad2/3 regulates the expression of target genes, including members of the zinc finger E‐box‐binding homeobox (ZEB) transcription factor family, by binding to Smad4 and translocating to the nucleus, thus contributing to lung cancer progression (90, 91).

The metastatic process is complex and involves interactions between cancer cells and surrounding tissues in the new microenvironment and cellular signaling within the cancer cells. Epithelial-mesenchymal transition (EMT) is a biological process in which epithelial cells lose polarity and intercellular adhesion. It is a key step in initiating cancer metastasis (92).In the study conducted by Karina, network analysis of 92 proteins exhibiting differential expression between CL16 cell transplant tumors with high miR-155 expression and control CL16 cell transplant tumors revealed three potential roles for miR-155. The protein expression of target genes ALDH1A1, PIR, and PDCD4 was reduced, indicating that miR-155 inhibits the expression of these proteins, which are known to be involved in metastasis formation. Consequently, these proteins may serve as effectors of altered miR-155 in our model, demonstrating that high expression of miR-155 in cancer cells inhibits extravasation and/or colonization during the later stages of the metastasis process (93). Current research indicates that the RASSF protein plays a significant role in tumor suppression and is involved in various critical biological functions, including proliferation, cell cycle regulation, apoptosis, and DNA repair (94). Notably, RASSF4 is widely expressed in normal tissues, whereas its expression is down-regulated in tumors, suggesting that RASSF4 functions as a tumor suppressor in a range of malignant tumors (95). In the study conducted by Li et al., it was found that exosomal miR-155 and miR-196a-5p secreted by M2-type tumor-associated macrophages (TAMs) in NSCLC metastasis promote NSCLC progression by regulating RASSF4 (92, 93).

#### 4.1.3 Role of MiR-155 in NSCLC chemotherapy resistance

In general, the efficacy of chemotherapeutic drugs is limited in the treatment of lung cancer, and the carcinoma cells may readily develop resistance to the drugs in clinical practice, significantly reducing the therapeutic efficacy of chemotherapy. Cisplatin is a chemotherapeutic agent that is commonly used for the treatment of lung cancers, but resistance to cisplatin and its severe toxic side effects limit its clinical application. The apoptotic protease activating factor 1 (Apaf-1) may be a target for miR-155. In the cytosol, Apaf-1 can bind with cytochrome c released from the mitochondrial inter-membrane and activate the initiator caspase-9, eventually resulting in cellular apoptosis. BCL2-associated X protein (Bax) is an important factor in destabilizing mitochondrial integrity, serving as an essential gateway to mitochondrial dysfunction and activation of the intrinsic apoptotic pathway (96). Zang et al. postulated that the deregulation of miR-155 expression could modulate the Apaf-1-related mitochondrial apoptotic pathway and enhance the sensitivity of lung cancer cells to cisplatin treatment (97). Doxorubicin targets DNA and topoisomerase II (Topo I I) to inhibit DNA synthesis and transcription, arrest tumor cell growth, and induce apoptosis. Clinical studies have found that doxorubicin, in combination with other chemotherapeutic drugs, acts as a treatment for lung carcinoma. LV et al. found that miR-155 suppression inhibited the activation of AKT and extracellular signal-regulated kinase (98). The transcriptional activity of nuclear factor-KB and activator protein-1 were also downregulated Arsenic trioxide (AS203, ATO) has been successfully used in the treatment of relapsed/refractory acute promyelocytic leukemia (APL) since the 1970s. Subsequent research by Alice et al. indicated that ATO is cytotoxic to lung cancer cells, with its biological activity linked to oxidative damage, alterations in cell morphology, and apoptosis. In 2017, Gu et al. demonstrated that miR-155 mediates the Nuclear Factor Erythroid 2-related Factor 2(NRF2) signaling pathway by upregulating resistance to ATO in lung cancer cells; however, its downregulation can lead to increased cell apoptosis (99–101). Additionally, miR-155 is essential in resistance to targeted therapies, such as the EGFR tyrosine kinase inhibitor (TKI) gefitinib, used to treat lung cancers with activating EGFR mutations. It induces gefitinib resistance by targeting Forkhead Box O3A (FOXO3A) (102, 103). Current research indicates that miR-155 promotes resistance to chemotherapy drugs and can mitigate this resistance when treated with miR-155 inhibitors. This drug resistance is mediated by a novel TP53/miR-155 feedback loop, suggesting that miR-155-targeted therapies hold promise for overcoming the development of drug resistance (104). Notably, the overexpression of miR-155 in tumor cells has emerged as a significant contributor to chemotherapy resistance and is linked to increased invasiveness and poor prognosis in NSCLC. These findings underscore the pivotal role of miR-155 in promoting chemoresistance, highlighting the necessity for targeted strategies in cancer therapy to address this challenge. Furthermore, given that high levels of miR-155 are associated with resistance to chemotherapy and radiotherapy, the therapeutic downregulation of miR-155 is warranted. The current study demonstrates that the overexpression of miR-155 significantly enhances glucose metabolism regulated by hexokinase 2(HK2). Significantly, the inhibition of miR-155 sensitizes lung cancer cells to radiation by disrupting glucose metabolism, thereby clarifying the role of miR-155 in regulating the radiosensitivity of NSCLC cells. In this context, miR-155 inhibitors may represent a promising therapeutic approach to enhance tumor sensitivity to chemotherapy and radiotherapy (105, 106).

#### 4.1.4 Role of MiR-155 in NSCLC immune response

One of the most extensively studied microRNAs in tumors and the immune system is miR-155. Research indicates that it plays a complex and significant role in the immune response associated with lung cancer. miR-155 not only enhances the anti-tumor immune response but also regulates the tumor microenvironment by influencing immune escape mechanisms, thereby contributing to the initiation and progression of tumors (107). The anti-tumor immune response refers to the process by which the body’s immune system recognizes and eliminates tumor cells through a series of immune cells, molecules, and mechanisms. miR-155 can significantly influence immune cells by targeting key regulatory molecules and transcription factors that modulate the immune system, thereby enhancing anti-tumor immunity (108, 109). Among the various immune cell types, miR-155 primarily regulates innate and adaptive immunity mechanisms. Dendritic cells (DCs), as professional antigen-presenting cells, are a crucial link between innate and adaptive immunity due to their unique ability to activate naive T cells (110). *In vitro* studies have shown that DCs treated with miR-155-rich exosomes produce more IL-12 and IFN-γ, and these cytokines significantly contribute to the anti-tumor activity of DCs (111). In addition,miR-155 was reported to change a “cold tumor” into a “hot one” and thus sensitize the tumor for checkpoint blockade immunotherapy. Consequently, miR-155 may facilitate communication between tumors and immune cells, thereby influencing the immune editing process of tumors (112). The potential of miR-155 as a predictive biomarker for immunotherapy efficacy has been reported (113). The mechanism underlying this process may involve the potential of miR-155 to alter the tumor microenvironment, specifically the immune cells present within it. An example of this alteration is illustrated by the repolarization of tumor-infiltrating macrophages (TAMs) (114, 115). Other studies have demonstrated that encapsulating miR-155 within nanomaterials such as sPEG/GLC can repolarize tumor-associated macrophages (TAMs) into anti-tumor M1 macrophages. This finding supports the notion that the delivery mechanism of miR-155 represents a promising approach to cancer treatment (115). Immune evasion is an important hallmark of cancer, and a better understanding of this mechanism is essential for developing effective strategies against cancer. Programmed death ligand-1 (PD-L1), also known as B7-H1 or CD274, is expressed in T cells, B cells, dendritic cells, macrophages, and mesenchymal stem cells (116). PD-L1 is also one of the key molecules in mediating tumor immune evasion. PD-L1 expressed in cancer cells could specifically bind to the receptor (PD-1) on the surface of activated T cells in the tumor microenvironment and transmit negative regulatory signals to inhibit T cell activation, proliferation, or cytokine secretion (117–119). Studies have shown that miR-155-5p inhibits PD-L1 gene expression by directly binding to relevant sites in response to cytokine stimulation. This suggests that the interaction between miR-155 and PD-L1 may reveal a novel mechanism for inflammation-related tumorigenesis and highlights the potential therapeutic applications of miR-155-5p and PD-L1 in LUAD (120).

**Figure 4:**
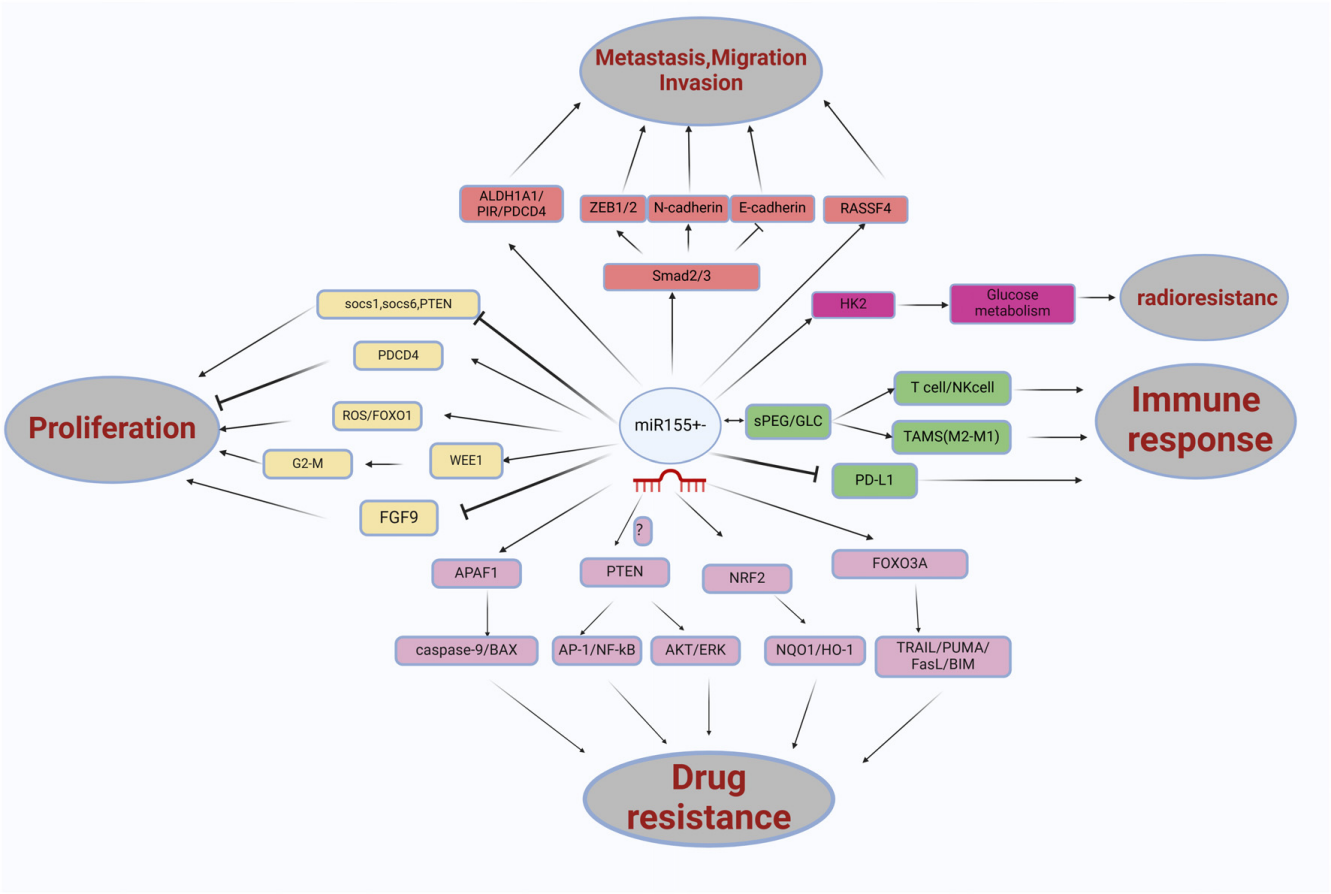
The role miRNA-155 in non-small cell lung cancer (NSCLC) is multifaceted, involving various pathways and interactions. miRNA-155 influences several key factors, including FOXO1/ROS, WEE1/G2/M, SOCS1/SOCS6/PTEN, PDCD4, FGF9, ALDH1A1/PIR/PDCD4, SMAD2/3, RASSF4, APAF1, NRF2, FOXO3A, PTEN, and nano-encapsulated materials such as sPEG/GLC. These interactions significantly affect glucose metabolism processes, contributing to the proliferation, migration, invasion, metastasis, drug resistance, and immune response in NSCLC.

In summary, the function of miR155 can be elucidated through its target genes (See **Figure 4**). Its expression is regulated by various proteins, primarily at the post-transcriptional level.

## 5 Clinical application potential of mircoRNA155

Numerous articles in cancer research have demonstrated the oncogenicity of miR-155. Some studies indicate that miR-155 is essential for activating immune responses, suggesting that inhibiting miR-155 in tumor-associated immune cells may promote tumor immune evasion and enhance tumor growth rather than suppressing it. This notion is supported by evidence showing that the loss of miR-155 in cells within the tumor microenvironment facilitates tumor growth (121, 122). Conversely, many studies have established that cancer cells generate sufficient energy for proliferation through increased glycolysis and mitochondrial dysfunction, known as the Warburg effect. Elevated levels of glycolysis in cancer cells have been linked to resistance to chemotherapy and radiotherapy (123). Furthermore, radiation has been shown to induce aerobic glycolysis via reactive oxygen species (124). Relevant studies indicate that the inhibition of miR-155 reduces glycolysis, ultimately rendering NSCLC cells more sensitive to radiation. Consequently, miR-155 may be a promising therapeutic target for developing anti-cancer drugs (106, 125). Hou et al.’s study demonstrated that miR-155 promotes the proliferation of NSCLC cells by inhibiting FoxO1 and increasing reactive oxygen species (ROS) production. This finding underscores the miR-155/FoxO1/ROS axis as a novel therapeutic target for inhibiting NSCLC growth. Compared to traditional small molecule drugs, miRNA and mRNA-targeting oligonucleotides offer several advantages, the most significant being their capacity for chemical modification to enhance pharmacodynamic and pharmacokinetic profiles and their ability to target multiple genes (126) simultaneously. Immunotherapy represents a promising approach for utilizing miR-155 in tumor treatment. Among various strategies, active immunotherapy based on dendritic cells (DCs) is one of the most promising current treatments. Research conducted by Hodge et al. indicated that the overexpression of miR-155 in DCs enhances their capacity to stimulate CD8+ T cell anti-tumor responses (127). Additionally, a study demonstrated that delivering a miR-155 mimic to dendritic cells within the tumor microenvironment of orthotopic ovarian cancer xenografts activated anti-tumor immune responses, inhibited tumor growth, and improved mouse survival (128). Other studies suggest that miR-155 should be down-regulated during treatment, as elevated levels of miR-155 have been linked to cancer resistance to chemotherapy and radiotherapy. In this context, miR-155 inhibitors may represent a novel therapeutic approach to enhance tumor sensitivity to these treatments while also reshaping the tumor’s immune microenvironment, increasing its susceptibility to the immune system and its resistance to immunotherapy (105). In November 2015, a phase I clinical trial was initiated to evaluate the LNA-modified anti-miR-155 antibody MRG-106, which further supports the potential effectiveness of miRNA inhibition in clinical applications and may inspire future developments of anti-miR-155 antibodies for cancer treatment (81). Importantly, given the favorable safety of cobomarsen, a miR-155 inhibitor, reported in phase 1 clinical trial (77). Considering the favorable safety profile reported in phase 1 clinical trials of the miR-155 inhibitor cobomarsen, a subsequent study conducted by researchers from the Department of Medicine at Houston Methodist Research Institute in the United States built upon previous findings published in Cancer. By establishing a multi-scale mechanistic model and calibrating it with *in vivo* data, the study extrapolated its results to humans, revealing that anti-miR-155 delivered via nanoparticles holds promise for treating non-small cell lung cancer. The findings are detailed: First, in monotherapy, anti-miR-155 administered at 2.5 mg/kg every three weeks demonstrated significant anti-cancer activity, with a median progression-free survival of 6.7 months. Second, in combination therapy, the two-drug regimen of anti-miR-155 and cisplatin resulted in a median progression-free survival of 11.3 months. Furthermore, the three-drug combination therapy comprising anti-miR-155, cisplatin, and pembrolizumab achieved a median progression-free survival of 13.1 months. Notably, the latter combination regimen is regarded as more practical due to its simplicity and cost-effectiveness (47). These promising, though early, results with Cobomarsen highlight the value of miR-155 as a therapeutic target and support further research on miR-155 in other diseases and other miRNAs in cancer. Expanded anti-miR-155 therapies could focus on targeting miR-155 in stromal cells of tumors to reduce inflammation in different malignancies. Meanwhile, applying the optimized backbone chemistry from the anti-miR-155 molecule to other anti-miR molecules may accelerate the development of future therapeutics targeting miRNAs.

## 6 Delivery mechanism of anti-mircoRNA155

Preclinical studies indicate that microRNAs (miRNAs) possess significant therapeutic potential in cancer management. However, the silencing of abnormally expressed miRNAs *in vivo* can be achieved through various nucleic acid analogs, including locked nucleic acids (LNA),2’-O-methyl oligonucleotides (such as antagomiRs), and peptide nucleic acids (PNAs) or nanoencapsulated PNAs. As with most RNA-based therapeutics, these strategies face challenges related to nonspecific organ biodistribution, reticuloendothelial (RES) clearance, and endolysosomal trafficking (129, 130). In a study, they introduce a novel antimiR delivery platform designed to target the acidic tumor microenvironment, evade systemic clearance by the liver, and facilitate cellular entry through non-endocytic pathways. Our findings indicate that linking peptide nucleic acid (PNA) antimiRs to a peptide featuring a low pH-induced transmembrane structure (PHLIP) results in a unique tumor-targetable construct under acidic conditions, such as those found in solid tumors (pH ~ 6). This approach enables antimiRs to effectively cross the plasma membrane and inhibit the miR-155 oncomiR in mouse models of lymphoma (131). Among them, a recent *in vivo* study showed that we used a 1,2-dioleoyl-sn-glycero-3phosphocholine (DOPC) liposomal nanoparticle as a delivery vehicle for anti-miR-155 for the treatment of lung cancer, We showed that anti-miR-155-DOPC significantly reduces miR-155 expression in lung tumors and that it resensitizes chemoresistant tumors to chemotherapeutic agents, such as cisplatin. Even though DOPC is used for systemic delivery, we found that it did not induce the immune system or any toxicities *in vivo* in mice. DOPC nanoparticles containing siRNA against Ephrin A2 (EphA2) are currently being evaluated in a phase I clinical trial for the treatment of advanced, recurrent cancers and seem to have a favorable toxicity profile so far (ClinicalTrials.gov Identified NCT01591356) (104). Based on current research, we summarize the delivery methods of anti-miR-155. Specifically, there are two primary types: local delivery and systemic delivery. Local delivery may be a viable strategy for certain localized tumors, such as lung cancer and skin cancer, as it can reduce systemic toxicity and enhance drug concentration at the tumor site. This method includes the use of nanoparticles, liposomes, and local injections. Conversely, systemic delivery may be more appropriate for tumors that are difficult to locate or for cases involving multiple tumors. This approach encompasses drug-loaded nanoparticles, viral vectors, and antibody-drug conjugates (ADCs), among others.

## 7 Discussion

Currently, despite an increased understanding of lung cancer and improvements in diagnosis and treatment methods, the disease continues to exhibit a high mortality rate and poor prognosis due to its aggressive nature. As researchers delve deeper into the study of miR-155, alterations in the miRNA sequence may emerge as critical factors in the pathogenesis of various tumors, including lung cancer(See **Table 2**). Furthermore, the production and expression of several miRNAs in cancer cells are significantly distinct from those in normal cells, indicating that these molecules may contribute to tumor growth, angiogenesis, and immune evasion (135). MiRNAs regulate numerous intracellular signaling pathways and elicit various effects, and they can be categorized based on their influence on tumor suppressor genes and oncogenic miRNAs (oncomirs) (10, 136). Notably, miR-200b-3p has been found to enhance the proliferation and metastasis of cancer cells (137). Additionally, miR-126 and miR-484 function as oncogenes that facilitate the progression of non-small cell lung cancer (138, 139). Conversely, the downregulation of miR-16-5p, which is a tumor suppressor, increases cancer signaling activity (140). In the context of cancer, the role of miRNAs as oncogenes or tumor suppressors is contingent upon the target genes they regulate. For instance, miR-21 is recognized as an oncogenic miRNA that targets tumor suppressor genes such as PTEN and PDCD4 (141), while miR-34a is another oncogenic miRNA that targets oncogenes such as MYC and BCL2 (142). Additionally, miRNAs can enhance cancer cell proliferation and survival by targeting cell cycle regulation, apoptosis, and DNA damage response genes. For instance, the miR-17-92 gene cluster, frequently upregulated in various cancers, promotes cancer cell proliferation by inhibiting the tumor suppressor gene PTEN (143). Furthermore, specific miRNAs have been demonstrated to regulate epithelial-to-mesenchymal transition (EMT) by targeting genes involved in cell-cell adhesion and cytoskeletal organization (144). Specifically, miR-200 family miRNAs inhibit EMT by targeting the transcription factors E box-binding zinc finger protein 1 (ZEB1) and ZEB2 (145). Similarly, miRNAs have been implicated in regulating angiogenesis by targeting genes that are part of angiogenic signaling pathways. For example, miR-126 inhibits angiogenesis by targeting vascular endothelial growth factor A (VEGF-A) and phosphatidylinositol 3-kinase regulatory subunit 2 (PIK3R2) (146). Moreover, miRNAs can also influence immune responses by targeting genes involved in immune signaling pathways. For instance, miR-155 promotes inflammation by targeting the negative regulators of nuclear factor-κB (NF-κB) and suppressor of cytokine signaling 1 (SOCS1) (147, 148). In addition to targeting gene regulation and immunotherapy, miRNA-targeted therapy encompasses various other aspects. For instance, Liu et al. investigated the application of miRNAs to reprogram tumor-associated macrophages (TAMs). Polarizing these macrophages into M1 macrophages, which possess anti-tumor properties, presents a viable approach for cancer treatment (115). Furthermore, Cubillos-Ruiz et al. intravenously administered miR-155 mimic RNA into tumor-associated dendritic cells (DCs) *in vivo* (128), demonstrating its ability to inhibit the progression of established ovarian cancer. They propose that enhancing miR-155 levels in TAMs may offer a potential strategy to repolarize these macrophages and mitigate immunosuppression within the tumor microenvironment, thereby hindering tumor progression. Similarly, Wang et al. (149) found that the overexpression of miR-155 in breast cancer cells significantly delays tumor progression by increasing the recruitment of effector immune cells to TME. However, Price et al. (150) argue that several biological challenges must be addressed to achieve effective TAM-targeted miRNA therapy: ① specifically targeting TAMs within the tumor microenvironment; ② promoting the uptake and cytoplasmic release of miRNA; and ③ preventing miRNA capture by tumors or other cells. Recently, Hussen et al. highlighted the potential of miRNA-targeted cancer treatment utilizing the CRISPR/Cas (CRISPR-associated protein, Cas) system (151). In cancer research, the CRISPR/Cas system has enhanced our understanding of the non-coding genome, tumor heterogeneity, and previously unrecognized challenges in precision medicine, opening new avenues for exploration. CRISPR/Cas-based gene-editing technology now enables precise and permanent mutation targeting, offering opportunities to target small non-coding RNAs such as microRNAs (miRNAs) (152, 153).

**Table 2:** Role of MiR-155 in NSCLC.

Target	Signaling pathways	Mechanism	Biological functions	Ref.
PDCD4	MiR-155→PDCD4	Downregulate PDCD4	Proliferation	(84)
FOXO1	MiR-155/FoxO1/ROS	Inhibits FoxO1 and increases ROS generation	Proliferation	(82)
WEE1	MiR-155→WEE1/G2-M	Block cell-cycle progression	Proliferation	(132)
FGF9	MiR-155→FGF9	Downregulate FGF9 cells	Proliferation	(85)
SOCS1,SOCS6 and PTEN	MiR-155→SOCS1,SOCS6 and PTEN	Downregulate SOCS1, SOCS6, and PTEN.	Proliferation	(133)
RASSF4	MiR-155→RASSF4	Downregulate in NSCLC tissues	Metastatic	(134)
SMAD2,3	MiR-155→Smad2,3 →ZEB1,2/N-cadherin/E-cadherin	Suppresses lung adenocarcinoma A549 cells	Migration and invasion	(89)
ALDH1A1/PIR/PDCD4	MiR155→ALDH1A1/PIR/PDCD4	Downregulate the three metastasis-associated proteins	Metastatic	(93)
HK2	Glucose metabolism	Overexpress miR-155	Radioresistance	(75, 76)
APAF1	MiR -155/Apaf - 1/caspase - 9/Bax	Enhance the sensitivity of lung cancer cells	Drug resistance	(97)
NRF2	HO-1/NQO1/Bax	Overexpress miR-155 mimic	Drug resistance	(101)
FOXO3A	TRAIL/PUMA/FasL/BIM	Suppresses cancer stemness of lung cancer	Drug resistance	(102, 103)
PTEN?	AP-1/NF-KB AKT/ERK	Silencing miR-155 reverses drug resistance	Drug resistance	(102)
sPEG/GLC	T cell/NK cell TAMS(M2-M1)	Repolarize TAMs to M1 macrophages *in situ* and induce tumor regression.	Immune response	(115)
PD-L1		Downregulate the expression of PD-L1.	Immune response	(120)

In the ongoing discussion regarding the significant controversies and unanswered questions in the field of miR-155 research, we summarize the discourse from several key perspectives. First, concerning the role of miR-155 in various diseases: Although miR-155 is believed to play a crucial role in immune response and tumorigenesis, its specific function in cancer remains contentious. For instance, in particular cancer types, miR-155 acts as an oncogene; in others, it may serve as a tumor suppressor gene. This is summarized in our article, as illustrated in **Table 3**. Secondly, we address the relationship between miR-155 and immune response, particularly regarding anti-tumor immunity and immune evasion. Finally, based on the interaction between miR-155-regulated NSCLC and immune cells within the tumor microenvironment, we propose investigating an immunomodulatory nanocarrier targeting miR-155 for treating NSCLC. This vector carries immunomodulatory factors, such as cytokines that promote immune activation or small molecules that inhibit immunosuppressive signaling pathways. On the one hand, these nanocarriers accurately deliver immune regulatory factors to tumor sites, altering the tumor microenvironment’s immunosuppressive state and enhancing immune cells’ cytotoxic activity against lung cancer cells. On the other hand, by inhibiting the expression or activity of miR-155, they can directly influence the malignant biological behavior of lung cancer cells while indirectly modulating immune cell function.

**Table 3:** Role of MiR-155 in cancer.

Target	Signaling pathways	Biological functions	Cancer	Ref.
DMTF1	DMTF1-Arf-p53	Tumor promoter	Bladder cancer	(68)
RhoA-mRNA	CCL17-CCR4 axis	Tumor promoter	Colon cancer	(69)
3’-UTR of PTEN	PI3K/Akt pathway	Tumor promoter	Hepatocellular carcinoma	(70)
CDX1	MiR-155→CDX1	Tumor promoter	glioma	(71)
TP53INP1	MiR-155→TP53INP1	Tumor promoter	Cervical cancer	(73)
SOCS1	JAK/STAT	Tumor promoter	Breast cancer	(74)
SOCS1	STAT3 signaling pathway	Tumor suppressor	Gastric cancer	(75)
SOCS1	STAT3 signaling pathway	Tumor promoter	Pancreatic cancer	(76)
BACH1,PICALM,JARID2	JAK/STAT, PI3K/AKT and MAPK pathways	Tumor suppressor	Mycosis fungicides	(77)
PTEN	PI3K/AKT	Tumor promoter	Nasopharyngeal carcinoma	(78)

## 8 Conclusions and prospects

In recent years, the application of miR-155 in tumors and related diseases has emerged as a significant area of research. Dysregulation of gene expression is frequently observed across various types of cancer, significantly influencing cancer progression by modulating the expression of oncogenes and tumor suppressor genes. In non-small cell lung cancer (NSCLC), miR-155 interacts with multiple pathways and assumes diverse roles. Understanding the up or down-regulation of different miRNAs concerning disease stages may be crucial. Given that miR-155 plays a pivotal role in regulating the immune system and influencing anti-tumor responses through its effects on various immune cell populations, the role miR-155 in cancer therapy warrants reconsideration in light of emerging therapeutic strategies. The modulation of miR-155 should consider not only the disease’s severity, including tumor stage and grade, but also the immune signatures of both the tumor and the surrounding tissue induced by the disease or its treatment (154–156). Furthermore, the level of miR-155 could serve as a potential marker for assessing the prognosis of NSCLC and evaluating resistance to conventional therapies, thereby providing valuable insights for identifying new therapeutic targets. Given that miR-155 is a key regulator of immune responses in solid tumors, it impacts a variety of immune cells involved in anti-tumor responses, including T cells, natural killer cells, and myeloid cells such as dendritic cells (DCs) and myeloid-derived suppressor cells (MDSCs). Consequently, applying miR-155 in conjunction with immunotherapy may prove advantageous for treating NSCLC. Future research on miRNA in NSCLC should further investigate its effects on tumor angiogenesis, glucose, lipid metabolism, and the maintenance of cancer stem cells. Although studies have incorporated miR-155 into the development of tumor biomarkers and inhibitors with significant potential, these efforts remain in the early stages and warrant ongoing attention. Although the role of miR-155 in NSCLC and other types of cancer is substantial, it remains controversial. Firstly, miR-155 acts as a double-edged sword in tumor development, possessing the ability to both suppress tumors and promote cancer. Additionally, it plays a crucial role in the immune response; however, the specific regulatory mechanisms by which it influences immune cells and the resultant effects are not yet fully understood. The intricate relationships among inflammation, immunity, and cancer involving miR-155 have yet to be verified. Furthermore, given the numerous potential targets of miR-155, there is ongoing debate regarding its primary functional targets. For instance, some studies indicate that miR-155 can target PTEN and regulate pathways such as AP-1/NF-kB and AKT/ERK, thereby influencing tumor immune response. Whether these processes are also implicated in NSCLC necessitates further investigation.
